# D-Dimer Is a Predictive Factor of Cancer Therapeutics-Related Cardiac Dysfunction in Patients Treated With Cardiotoxic Chemotherapy

**DOI:** 10.3389/fcvm.2021.807754

**Published:** 2022-01-21

**Authors:** Masayoshi Oikawa, Daiki Yaegashi, Tetsuro Yokokawa, Tomofumi Misaka, Takamasa Sato, Takashi Kaneshiro, Atsushi Kobayashi, Akiomi Yoshihisa, Kazuhiko Nakazato, Takafumi Ishida, Yasuchika Takeishi

**Affiliations:** Department of Cardiovascular Medicine, Fukushima Medical University, Fukushima, Japan

**Keywords:** cardio-oncology, D-dimer, cancer therapeutics-related cardiac dysfunction, heart failure, troponin I

## Abstract

**Background:**

D-dimer is a sensitive biomarker for cancer-associated thrombosis, but little is known about its significance on cancer therapeutics-related cardiac dysfunction (CTRCD).

**Methods:**

Consecutive 169 patients planned for cardiotoxic chemotherapy were enrolled and followed up for 12 months. All patients underwent echocardiography and blood test at baseline and at 3-, 6-, and 12 months.

**Results:**

The patients were divided into two groups based on the level of D-dimer (>1.65 μg/ml or ≦ 1.65 μg/ml) at baseline before chemotherapy: high D-dimer group (*n* = 37) and low D-dimer group (*n* = 132). Left ventricular ejection fraction (LVEF) decreased at 3- and 6 months after chemotherapy in high D-dimer group [baseline, 65.2% (62.8–71.4%); 3 months, 62.9% (59.0–67.7%); 6 months, 63.1% (60.0–67.1%); 12 months, 63.3% (58.8–66.0%), *p* = 0.03], but no change was observed in low D-dimer group. The occurrence of CTRCD within the 12-month follow-up period was higher in the high D-dimer group than in the low D-dimer group (16.2 vs. 4.5%, *p* = 0.0146). Multivariable logistic regression analysis revealed that high D-dimer level at baseline was an independent predictor of the development of CTRCD [odds ratio 3.93, 95% CI (1.00–15.82), *p* = 0.047].

**Conclusion:**

We should pay more attention to elevated D-dimer levels not only as a sign of cancer-associated thrombosis but also the future occurrence of CTRCD.

## Introduction

Recent advances in the diagnosis and treatment of cancers improve its prognosis. However, anticancer drugs, namely, anthracyclines, monoclonal antibodies, tyrosine kinase inhibitors, etc., induce cardiac dysfunction, resulting in poor prognosis in cancer survivors (1). Several cardiac biomarkers and echocardiographic parameters, such as troponins, myeloperoxidase, interleukin-1β (IL-1β), Nucleotide-binding domain-like receptor family pyrin domain containing 3, and reduced global longitudinal strain, are proposed to detect the early phase of cancer therapeutics-related cardiac dysfunction (CTRCD) and prompt cardioprotective treatment can improve cardiac function (2–7). Although those parameters are useful, careful monitoring is required for all patients to detect early signs of CTRCD. Thus, a novel biomarker that identifies high-risk patients before chemotherapy is desirable to perform effective clinical monitoring.

D-dimer is a sensitive biomarker for cancer-associated thrombosis, but accumulating evidence suggests that pretreatment D-dimer can be used as a prognostic biomarker for patients with solid tumors (8). In cardiovascular fields, elevated D-dimer is associated with not only thromboembolic events but also heart failure mortality in heart failure patients with reduced and preserved ejection fraction (EF) (9, 10).

Although D-dimer is a promising biomarker in the cardio-oncology field, little is known about the relationship between D-dimer and CTRCD. The present study aimed to evaluate the predictive impact of D-dimer before chemotherapy on the development of CTRCD.

## Methods

### Study Subjects and Protocol

**Figure 1:**
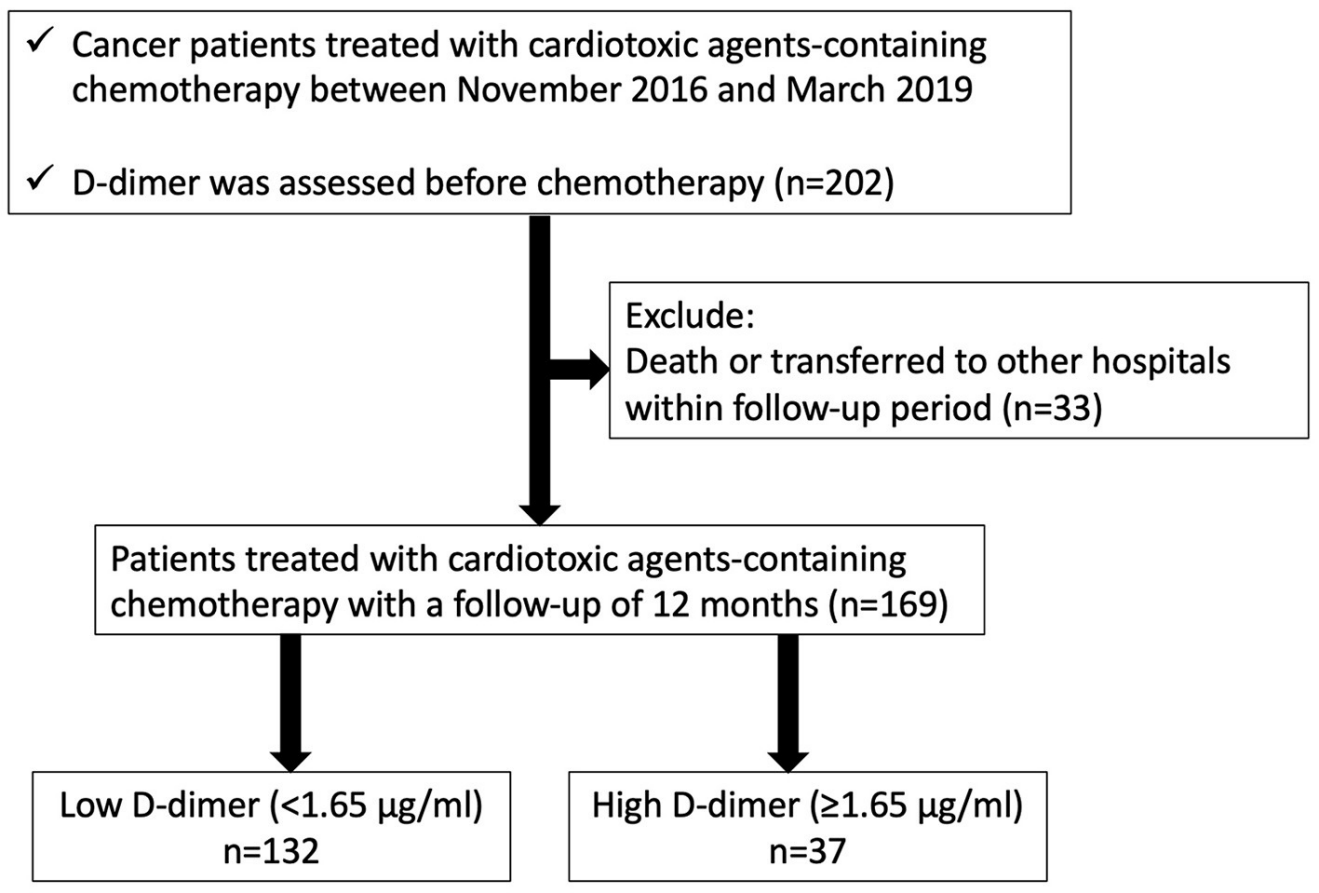
Patient cohort selection.

**Figure 2:**
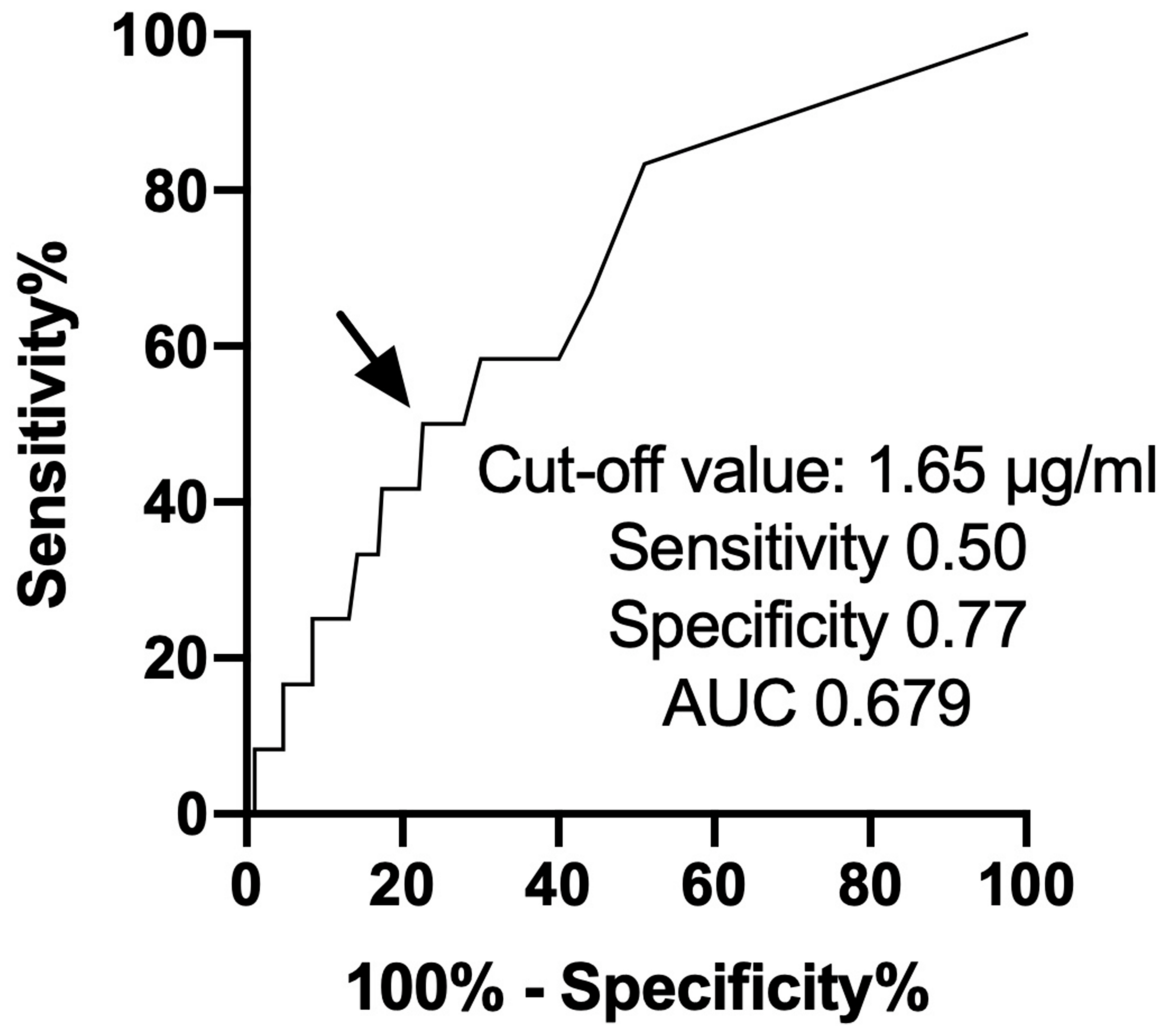
ROC curve analysis of D-dimer predicting the occurrence of cancer therapeutics-related cardiac dysfunction. ROC, receiver operator characteristic curve.

We enrolled 202 consecutive cancer patients, planned for cardiotoxic chemotherapy, such as anthracyclines, human epidermal growth factor receptor 2 (HER2) inhibitors, tyrosine kinase inhibitors, and proteasome inhibitors, at Fukushima Medical University hospital from November 2016 to March 2019 (Figure 1). Patients were excluded if they died or were transferred to other hospitals within 12 months follow-up period (*n* = 33). The remaining 169 patients were divided into two groups based on the cut-off value of D-dimer, which was defined by the receiver operator characteristic curve analysis to detect the occurrence of CTRCD (Figure 2).

Hypertension was defined as a history of use of an antihypertensive drug or systolic blood pressure of ≥140 mmHg and/or diastolic blood pressure ≥90 mmHg. Diabetes was defined as recent use of insulin treatment or hypoglycemic drug or hemoglobin A1c ≥6.5%. Dyslipidemia was defined as a history of use of cholesterol-lowering drugs, or triglyceride was ≥150 mg/dl, low-density lipoprotein cholesterol was ≥140 mg/dl, and/or high-density lipoprotein cholesterol was ≤ 40 mg/dl. A cumulative dose of anthracycline was expressed as a doxorubicin equivalent (1). HER2 inhibitors included trastuzumab and pertuzumab. Tyrosine kinase inhibitors included dabrafenib, trametinib, lenvatinib, sorafenib, dasatinib, bevacizumab, and pazopanib. Proteasome inhibitors included carfilzomib and bortezomib. Radiation therapy was defined as irradiation to the mediastinum and/or the heart field within the follow-up period. Transthoracic echocardiography and blood sampling test were performed at baseline as well as at 3, 6, and 12 months after the administration of cardiotoxic chemotherapy. All procedures used in this research were approved by the Ethical Committee of Fukushima Medical University.

### Echocardiography

Transthoracic echocardiography was performed by a trained sonographer, and images were checked by another trained sonographer and an echo-cardiologist. We measured cardiac function using EPIQ 7G (Philips Healthtech, Best, The Netherlands). Left ventricular (LV) EF was calculated using the modified Simpson's method according to the guideline from the American Society of Echocardiography and the European Association of Cardiovascular Imaging (11). The LV mass was calculated using the following formula:


$$\begin{array}{l} \text{Left}\;\text{ventricular}\left(\text{LV}\right)\text{ mass}\text{=0.8}\\ \times [1.04\times \{{\left(\begin{array}{l} \text{LV}\;\text{diastolic}\;\text{diameter}+\\ \text{interventricular}\;\text{septum}\;\text{wall}\\ \text{thicness}+\text{LV}\;\text{posterior}\;\text{wall}\;\text{thicness} \end{array}\right)}^{3}\\ -{\left(\text{LV}\;\text{diastolic}\;\text{diameter}\right)}^{3}\}]+0.6\text{g}\;(11). \end{array}$$

Cancer therapeutics-related cardiac dysfunction was defined as a decrease in EF by more than 10% points, to a value <53% (12). The LV end-diastolic volume index, LV end-systolic volume index, LV mass index, and left atrial volume index were measured using the B-mode ultrasound.

### Blood Sampling

High sensitivity cardiac troponin I (TnI) was measured using an assay based on Luminescent Oxygen Channeling Immunoassay technology and run on a Dimension EXL Integrated Chemistry System (Siemens Healthcare Diagnostics, Deerfield, IL, USA). B-type natriuretic peptide (BNP) levels were measured using a specific immunoradiometric assay (Shionoria BNP kit, Shionogi, Osaka, Japan). D-dimer was measured using a latex agglutination method (Lias Auto D-dimer Neo, Sysmex, Kobe, Japan).

### Statistical Analysis

All statistical analyses were performed using Prism 9 (GraphPad Software, San Diego, CA, USA) or R software packages version 3.6.3 (R core team 2020, Vienna, Austria). We used the Shapiro-Wilk test to discriminate which variables were normally or not normally distributed. Normally distributed variables were shown as mean ± SD. Non-normally distributed variables were indicated by a median with interquartile range. Category variables were shown in numbers and percentages. Student's *t*-test was used for variables following a normal distribution, the Mann-Whitney U-test was used for variables of the non-normal distribution, and the chi-square test was used for categorical variables. The time course of EF (baseline, 3-, 6-, and 12 months after the administration of anthracyclines) was evaluated using the Friedman test.

Logistic regression analysis was performed to identify the variables to predict the occurrence of CTRCD. We selected variables relating to the general condition and cardiac function, i.e., age, echocardiographic parameters, use of anthracyclines, BNP, hemoglobin, estimated glomerular filtration ratio, and the elevation of D-dimer. The variables presenting *p* < 0.05 in the univariable analysis were entered into the multivariable analysis. A receiver operating characteristic curve analysis was performed to determine the optimal cut-off value of the D-dimer for predicting the occurrence of CTRCD. The *p* of 0.05 or less was defined as significant.

## Results

First, we performed a receiver operating characteristic curve analysis to identify the threshold level of D-dimer to predict the occurrence of CTRCD (Figure 2). A total of 12 patients suffered from CTRCD within 12 months follow-up period. When we set the cut-off value of D-dimer at 1.65 μg/ml, sensitivity, specificity, and area under the curve to predict CTRCD were 50.0%, 80.3%, and 0.661, respectively. Then, we divided the patients into two groups based on the cut-off value. Table 1 shows patient characteristics at the baseline before chemotherapy. There were no statistical differences in age, sex, and the usage of angiotensin-converting enzyme inhibitors/angiotensin II receptor blockers and β-blockers. The high D-dimer group included a lower rate of breast cancer (35 vs. 67%, *p* = 0.0005), a higher rate of ovarian/uterine cancer (19 vs. 6%, *p* = 0.0151), and a higher rate of leukemia (16 vs. 4%, *p* = 0.0068) than low D-dimer group. Echocardiographic data demonstrated that EF was slightly higher in the high D-dimer group (67 ± 5 vs. 64 ± 5%, *p* = 0.0019). In laboratory data, the high D-dimer group showed lower hemoglobin values and higher BNP values.

**Table 1:** Baseline clinical characteristics of patients with elevated or non-elevated D-dimer.

**Variable**	**Entire cohort (*n* = 169)**	**Low D-dimer (*n* = 132)**	**High D-dimer (*n* = 37)**	***P*-value**
Age, years	57 ± 12	56 ± 12	58 ± 14	0.6265
Female, *n* (%)	146 (86%)	117 (89%)	29 (78%)	0.1078
**Medications**				
Use of ACEi or ARB	23	18	5	0.9846
Use of β-blockers	4	3	1	0.8791
**Cancer types**				
Breast cancer, *n* (%)	101 (60%)	88 (67%)	13 (35%)	0.0005
Lymphoma, *n* (%)	28 (17%)	20 (15%)	8 (22%)	0.3495
Ovarian or uterine cancer, *n* (%)	15 (9%)	8 (6%)	7 (19%)	0.0151
Leukemia, *n* (%)	11 (7%)	5 (4%)	6 (16%)	0.0068
Bone cancer, *n* (%)	2 (2%)	2 (2%)	0 (0%)	0.4513
Other cancers, *n* (%)	12 (7%)	9 (7%)	3 (8%)	0.7872
**Cancer therapy**				
Anthracyclines	138 (82%)	104 (79%)	34 (92%)	0.0687
HER2 inhibitors	36 (21%)	31 (23%)	5 (14%)	0.1905
Tyrosine kinase inhibitors	8 (5%)	6 (5%)	2 (5%)	0.8277
Proteasome inhibitors	5 (3%)	5 (4%)	0 (0%)	0.2295
Dose of anthracyclines (doxorubicin equivalent), mg/m^2^	200 [161–240]	200 [180–240]	180 [112–300]	0.3874
Radiation therapy, *n* (%)	20 (12%)	15 (11%)	5 (14%)	0.7205
**Cardiovascular risk factors**				
Hypertension, *n* (%)	40 (24%)	31 (24%)	9 (24%)	0.9154
Smoking history, *n* (%)	47 (28%)	37 (28%)	10 (27%)	0.9042
Diabetes mellitus, *n* (%)	16 (10%)	13 (10%)	3 (8%)	0.7493
Dyslipidemia, *n* (%)	44 (26%)	38 (29%)	6 (16%)	0.1235
**Echocardiographic parameter**				
LV end-diastolic volume index, mm/m^2^	45 [36–55]	45 [36–55]	46 [36–55]	0.6517
LV end-systolic volume index, mm/m^2^	15 [13–20]	15 [13–19]	16 [12–20]	0.6431
LV mass index, g/m^2^	70 [59–85]	70 [59–85]	75 [60–87]	0.4644
LA volume index, ml/m^2^	23 [17–30]	23 [17–28]	23 [19–32]	0.3159
LV ejection fraction, %	65 ± 5	64 ± 5	67 ± 5	0.0019
E/A	1.0 [0.8–1.2]	1.0 [0.8–1.2]	0.9 [0.8–1.1]	0.5788
**Laboratory data**				
Aspartate aminotransferase, IU/L	19 [15–23]	19 [16–23]	19 [15–26]	0.7973
Alanine aminotransferase, IU/L	15 [12–22]	15 [12–21]	15 [12–23]	0.7960
eGFR, ml/min/1.73 m^2^	72 [64–85]	73 [65–82]	69 [57–88]	0.3472
Hemoglobin, g/dl	13 [11–14]	13 [12–14]	11 [9–13]	0.0001
Uretic acid, mg/dl	4.7 ± 1.4	4.6 ± 1.3	4.7 ± 1.7	0.8197
B-type natriuretic peptide, pg/ml	12 [7–22]	11 [7–20]	17 [9–38]	0.0440
Troponin I, ng/ml	0.017 [0.017–0.017]	0.017 [0.017–0.017]	0.017 [0.017–0.017]	0.5440
D-dimer, μg/ml	0.6 [0.5–1.4]	0.5 [0.5–0.7]	3.1 [2.2–8.1]	<0.0001

*ACEi, angiotensin converting enzyme inhibitor; ARB, angiotensin II receptor blocker; HER2, human epidermal growth factor receptor 2; LV, left ventricular; E/A, early to late diastolic transmitral flow velocity; eGFR, estimated glomerular filtration ratio*.

**Figure 3:**
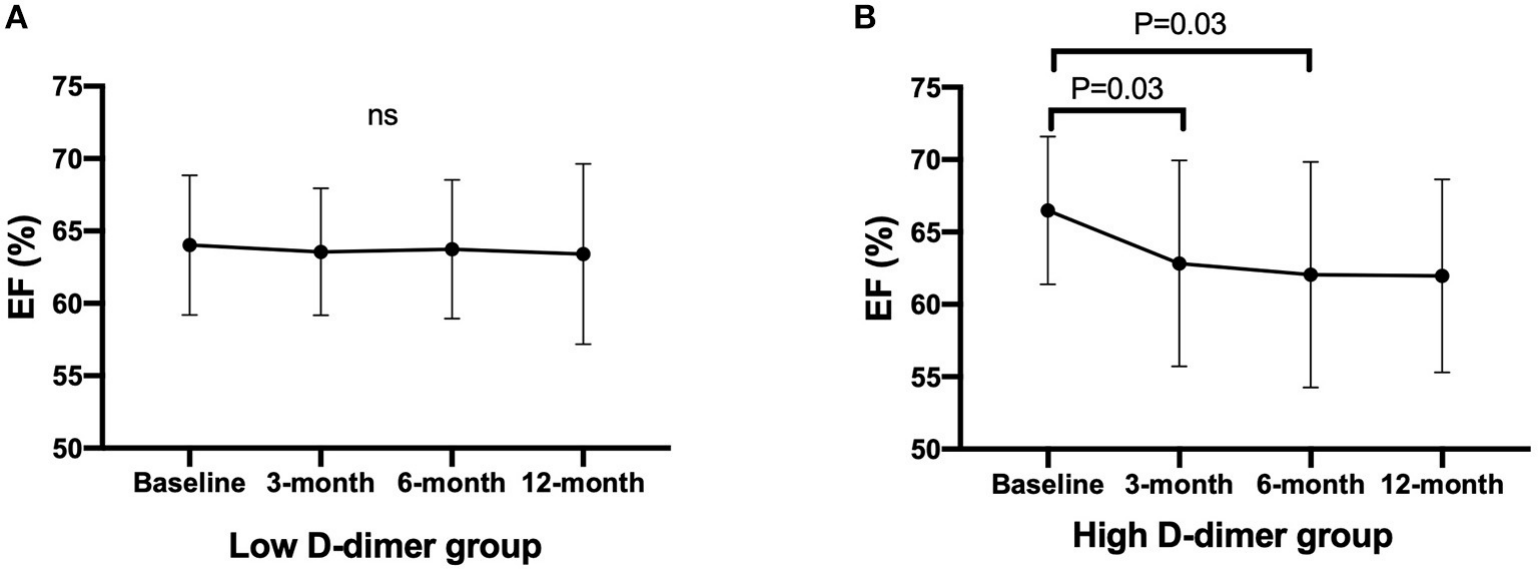
Time-dependent changes in EF after chemotherapy. Data are expressed in mean with SD. Statistics is performed using Friedman's test with Dunn's multiple comparisons test. EF, ejection fraction. **(A)** Changes in EF in the low D-dimer group. **(B)** Changes in EF in the high D-dimer group. EF, ejection fraction.

Time-dependent changes in EF are displayed in Figure 3. Low D-dimer group showed no changes in EF within the follow-up period, but EF was decreased at 3- and 6 months after chemotherapy in high D-dimer group [baseline, 65.2% (62.8–71.4%); 3 months, 62.9% (59.0–67.7%); 6 months, 63.1% (60.0–67.1%); 12 months, 63.3% (58.8–66.0%), *p* = 0.03, Figures 3A,B]. The reduction of EF from baseline was larger in high D-dimer group than in low D-dimer group (3 months: −4.0 ± 7.1 vs. −0.5 ± 5.3, *p* = 0.0015; 6 months: −4.8 ± 8.0 vs. −0.2 ± 6.2, *p* = 0.0004; 12 months: −4.5 ± 7.3 vs. −0.4 ± 6.6%, *p* = 0.0024).

The occurrence of CTRCD during the 12-month follow-up period was higher in the high D-dimer group than in the low D-dimer group (16.2 vs. 4.5%, *p* = 0.0146). Multivariable logistic regression analysis revealed that LV end-diastolic volume index [odds ratio 0.95, 95% CI (0.91–0.99), *p* = 0.0122] and high D-dimer levels [odds ratio 3.93, 95% CI (1.00–15.82), *p* = 0.0469] before chemotherapy were independent predictors of the development of CTRCD (Table 2).

**Table 2:** Parameters associated with the occurrence of CTRCD.

	**Univariate**		**Multivariate**	
	**OR (95% CI)**	***P*-value**	**OR (95% CI)**	***P*-value**
Age, per 1 year increase	1.01 (0.96–1.05)	0.8470		
Male	1.79 (0.32–33.57)	0.5852		
Use of anthracyclines	1.18 (0.29–7.94)	0.8353		
BNP, per 1 pg/ml increase	0.99 (0.98–1.02)	0.4239		
LV ejectioin fraction, per 1% increase	1.07 (0.95–1.22)	0.2591		
LV end-diastolic volume index, per 1 ml/m^2^ increase	0.95 (0.91–0.99)	0.0099	0.95 (0.91–0.99)	0.0122
E/A, per 1 increase	0.24 (0.05–1.39)	0.0934		
Left atrial volume index, per 1 ml/m^2^ increase	0.99 (0.94–1.05)	0.7157		
Hemoglobin, per 1 g/dl increase	1.13 (0.85–1.45)	0.3539		
Estimated GFR, per 1 ml/min/1.73 m^2^	0.98 (0.95–1.02)	0.2989		
Elevated D-dimer (1.65 mg/dl)	4.07 (1.20–13.84)	0.0218	3.93 (1.00–15.82)	0.0469

*CTRCD, cancer therapeutics-related cardiac dysfunction; BNP, B-type natriuretic peptide; E/A, early to late diastolic transmitral flow velocity; LV, left ventricular; GFR, glomerular filtration ratio*.

## Discussion

In the present study, we revealed the predictive features of D-dimer in patients treated with cardiotoxic agents. First, the threshold level of D-dimer was 1.65 μg/ml to predict the development of CTRCD. Second, EF was decreased time-dependently in high D-dimer patients. Third, the occurrence of CTRCD was significantly higher in high D-dimer patients.

D-dimer is a pivotal biomarker of hypercoagulability and thrombosis. Fibrin-bound plasmin degrades the fibrin network into soluble fragments D-dimers and E fragments, thus increased levels of D-dimer represent a global activation of coagulation and fibrinolysis (13). Cancers produce hypercoagulable and prothrombotic situations by secreting several pro-thromboembolic factors, such as mucins, cysteine protease, and tissue factors (14). Therefore, thrombi are easily generated in patients with cancer, and thromboembolism is the second leading cause of cancer-related morbidity and mortality (15, 16). Although D-dimer is an established and widely used biomarker for the screening of thrombus formation in patients with cancer, prognostic features of D-dimer have become clinically overt recently. The link between D-dimer and cancer progression is reported in several papers (17, 18), and higher levels of D-dimer are associated with poor prognosis in cancer patients (18). Although the precise mechanisms are still complex and uncovered, the pro-coagulable state may produce a suitable milieu for cancer progression by recruitment of pro-metastatic leukocytes, adhesion to the endothelium, transendothelial migration, and restriction in natural killer cell-mediated clearance of micrometastasis (19, 20). Accumulating evidence showed that abnormal inflammation and oxidative stress are key factors to the development of heart failure, and these also play important roles in cancer progression and thrombus formation (21–25). For example, IL-1β, a representative inflammatory cytokine, induces cardiac dysfunction and thrombus formation (26). IL-1β activates myddosome complex, such as nuclear factor κB, myeloid differentiation factor 88, cryopyrin, and p38-MAPK, in cardiomyocytes, leading to dysregulates metabolism in the sarcoplasmic reticulum, calcium homeostasis, and myocardial apoptosis and necrosis (7). In addition, IL-1β increased pro-coagulant state through activating tissue factor-dependent mechanisms in endothelial cells (27). Gomes et al. reported that blockade of IL-1 receptor abolished the neutrophil extracellular traps-dependent pro-thrombotic state and attenuated cancer-associated thrombosis in murine breast cancer model (25). Considering the fact that inflammation is a major contributor to cardiac dysfunction and thrombus formation, cancer patients with high D-dimer may be predisposed to cardiac dysfunction due to a chronic inflammatory state. Cardiotoxic chemotherapeutic agents are crucial and indispensable to performing cancer treatment. Anthracyclines induce pro-inflammatory responses by increasing tumor necrosis factor α (TNF-α), IL-1β, and IL-6, leading to tumor cell death (28). Not only anthracyclines but also targeted chemotherapy, such as trastuzumab and bevacizumab, increased inflammatory cytokines after the treatment (29, 30). In the present study, the patients with a high D-dimer group may already have been exposed to an inflammatory state before chemotherapy and were vulnerable to additional inflammatory stress by cardiotoxic agents, resulting in the development of CTRCD. To elucidate the precise mechanisms was beyond this study, but the importance of D-dimer should be noted in the cardio-oncology field. Intervention with Pravastatin in Ischemic Disease (LIPID) study revealed that elevated D-dimer levels predict long-term risk of arterial and venous events, cardiovascular disease mortality, in addition to that, increased cancer incidence and mortality rate (31). To the best of our knowledge, this is the first report assessing the relationship between D-dimer levels and the development of CTRCD. The importance of D-dimer should be taken into account when managing patients with cancer who are treated with cardiotoxic chemotherapy.

## Limitation

This study has several limitations. First, this study was performed using a relatively small number of patients and a short follow-up period by a single center. Slight differences in EF at baseline may be due to the small sample size of the high D-dimer group. Second, although not statistically significant, a higher proportion of patients in the high D-dimer group received anthracycline-containing chemotherapies. This might affect the results in the reduction in EF in the high D-dimer group. Longer follow-up and larger population data were needed to confirm the importance of D-dimer to the development of CTRCD and cardiovascular prognosis. Third, D-dimer has modest sensitivity and specificity to predict CTRCD in the present study. The mechanisms by which CTRCD development must be complicated, thereby predicting CTRCD by a single biomarker is still challenging. D-dimer is frequently analyzed in daily clinical practice to detect cancer-associated thrombosis. Therefore, we think D-dimer is easy and useful for predicting both CTRCD and thrombus formation.

## Conclusion

Elevated D-dimer is a pivotal biomarker to predict CTRCD. D-dimer should be taken into account when managing cancer patients treated with cardiotoxic chemotherapy.

## Data Availability Statement

The raw data supporting the conclusions of this article will be made available by the authors, without undue reservation.

## Ethics Statement

The studies involving human participants were reviewed and approved by Ethical Committee of Fukushima Medical University Hospital. The patients/participants provided their written informed consent to participate in this study.

## Author Contributions

MO created the study design, analyzed the data, and drafted the manuscript. DY created the study design and analyzed the data. TM, TS, TK, and AK acquired the data. AY, KN, TI, and YT interpreted the data and revised the manuscript. All authors contributed to the conception, design, critical revision, and final approval of this manuscript.

## Funding

JSPS KAKENHI (Grant Number JP20K08493).

## Conflict of Interest

The authors declare that the research was conducted in the absence of any commercial or financial relationships that could be construed as a potential conflict of interest.

## Publisher's Note

All claims expressed in this article are solely those of the authors and do not necessarily represent those of their affiliated organizations, or those of the publisher, the editors and the reviewers. Any product that may be evaluated in this article, or claim that may be made by its manufacturer, is not guaranteed or endorsed by the publisher.
